# Acute, Painless Monocular Vision Loss: Non-arteritic Ischemic Optic Neuropathy Associated With Untreated Obstructive Sleep Apnea, Uncontrolled Type 2 Diabetes Mellitus, Essential Hypertension, and Hyperlipidemia: A Case Report

**DOI:** 10.7759/cureus.26687

**Published:** 2022-07-09

**Authors:** Raied Hufdhi, Arjun Chandra, Farshid Bozorgnia, Hadeel Rushdi, Abdulaziz Aldhafeeri, Nezam Altorok

**Affiliations:** 1 Internal Medicine, University of Toledo, Toledo, USA; 2 Rheumatology, University of Toledo, Toledo, USA

**Keywords:** diabetes mellitus, hypertension, obstructive sleep apnea, giant cell arteritis, aion, arteritic ischemic optic neuropathy, naion, non-arteritic ischemic optic neuropathy

## Abstract

Non-arteritic ischemic optic neuropathy (NAION) is a common cause of acute, painless monocular vision loss in adults older than 50. NAION is a diagnosis of exclusion established once arteritic disease and other etiologies of acute vision loss have been ruled out. Clinicians need to distinguish NAION from arteritic ischemic optic neuropathy (AION) since failing to appropriately treat patients presenting with AION results in an inferior prognosis. NAION is often associated with risk factors like obstructive sleep apnea, atherosclerosis, diabetes mellitus, hypertension, hyperlipidemia, smoking, and phosphodiesterase-5 inhibitors. Clinicians need to address these risk factors to help prevent the development of NAION in their patients. Here, we present the case of a 63-year-old Caucasian male who presented with acute, painless monocular vision loss.

## Introduction

Non-arteritic ischemic optic neuropathy (NAION) is a disease characterized by acute, painless monocular vision loss that represents one of the most common causes of optic neuropathy in adults older than 50 [1]. The exact pathophysiology of NAION is unclear, but it appears to be caused by decreased perfusion to the optic nerve [1,2]. It is essential to distinguish NAION from arteritic ischemic optic neuropathy (AION). Although both conditions present acute vision loss, AION has a much worse prognosis than NAION. Given that AION has an extremely poor prognosis if not treated promptly and appropriately, clinicians need to understand the clinical features of NAION that distinguish it from AION [1]. Here, we present the case of a 63-year-old Caucasian male who presented with acute, painless monocular vision loss.

## Case presentation

**Figure 1:**
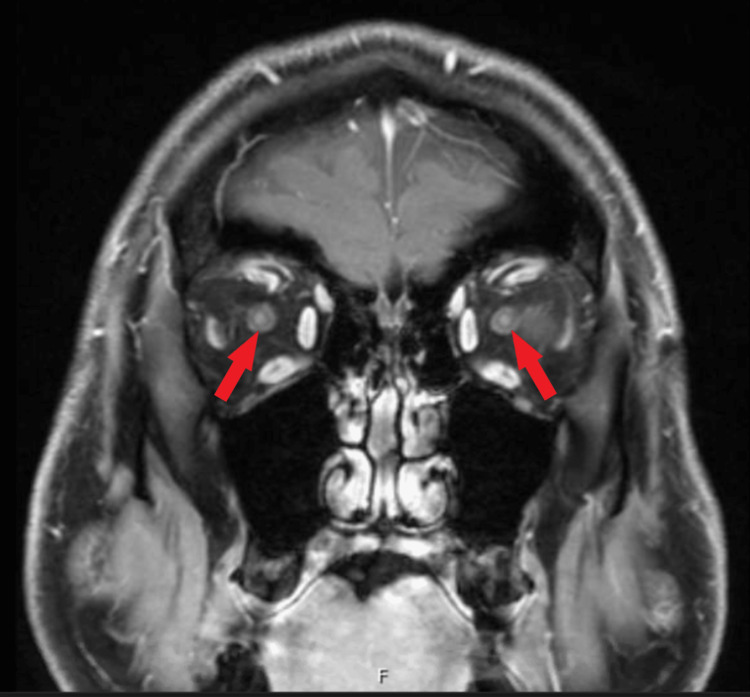
MRI of the orbits with contrast showing subtle uniform enhancement of the left and right optic nerves immediately behind the globes (arrows)

A 63-year-old Caucasian male presented with an acute, painless loss of vision affecting his right eye that occurred without any identifiable inciting event. He complained of blurry vision isolated to his right eye without any other neurological symptoms. The initial ophthalmological evaluation suggested ischemic optic neuropathy. The patient did not report jaw claudication, scalp tenderness, or temporal headache. Physical exam was positive for right-sided decreased visual acuity, diminished visual field, 360 degrees optic nerve edema and obscuration of vessels, optic disc hemorrhage, and 1+ optic disc pallor. Visual acuity of the right eye with correction was reported as HM (hand motion) 2’ while visual acuity of the left eye with correction was reported as 20/20. There was no afferent pupillary defect in either eye. Magnetic resonance imaging (MRI) of the brain with and without contrast was unremarkable. MRI of the orbits with contrast showed subtle uniform enhancement of the left and right optic nerves immediately behind the globes (Figure 1).

The patient was started on high-dose intravenous steroids and evaluated by ophthalmology, rheumatology, and vascular surgery. Complete blood count and comprehensive metabolic panel were unremarkable. Erythrocyte sedimentation rate (ESR) and C-reactive protein (CRP), measured before the administration of steroids, were 28 mm/h and 0.3 mg/dL, respectively. The lipid panel was remarkable for elevated low-density lipoprotein levels of 155 mg/dL, an elevated total cholesterol level of 314 mg/dL, and an elevated triglyceride level of 531 mg/dL. Hemoglobin A1c was 12.1%. Temporal artery ultrasound, performed before the administration of steroids, did not show evidence of giant cell arteritis (GCA). Temporal artery biopsy, performed after the administration of steroids, was also negative for GCA. Since a diagnosis of NAION was considered very likely, steroids were discontinued. After being discharged, the patient was seen in the rheumatology clinic. He underwent polysomnography, which revealed severe obstructive sleep apnea (OSA) with an apnea-hypopnea index (AHI) of 33.2 events per hour, thus requiring treatment with continuous positive airway pressure (CPAP) therapy. Although he denied using phosphodiesterase-5 (PDE-5) inhibitors prior to his hospital admission, he had multiple other risk factors for NAION, including hypertension, hyperlipidemia, uncontrolled type two diabetes mellitus, and untreated OSA.

## Discussion

NAION is characterized by acute, painless monocular vision loss. No definitive treatment for NAION is available; therefore, it is essential to identify and control modifiable risk factors to prevent this condition. NAION is thought to occur because of decreased perfusion to the optic nerve and has been associated with conditions that may cause decreased optic nerve perfusion via microvascular occlusions, such as diabetes mellitus and hyperlipidemia. Additionally, NAION may occur due to impairment of the optic nerve head's autoregulatory mechanisms by factors such as arteriosclerosis or chronic arterial hypertension [2,3]. Based on angiographic evidence, NAION is not associated with embolic or thrombotic events. NAION is a diagnosis of exclusion and is established after ruling out arteritic disease and other etiologies of acute vision loss. It is essential to rule out AION, which presents similarly to NAION but has a much worse prognosis than NAION if not treated promptly and appropriately. One significant feature distinguishing NAION from AION is optic disc edema with hyperemia compared to the significant optic disc pallor seen in AION. It is important when obtaining a history from patients presenting with acute, painless monocular vision loss to inquire if they have experienced symptoms like jaw claudication, temporal headache, scalp tenderness, weight loss, or fatigue since such symptoms should alert clinicians to the more urgent diagnosis of AION secondary to GCA.

Additionally, evaluation of these patients should include ESR and CRP since the elevation of these inflammatory markers points more toward diagnosing AION than NAION [1]. Finally, performing a temporal artery biopsy or temporal artery ultrasound should be considered in these patients. While negative results for GCA do not rule out AION, such results would place the diagnosis of AION much lower on the differential [1,4]. A summary of GCA's clinical and laboratory features and the frequencies in which they occur is presented in Table 1 [4]. Failing to distinguish AION from NAION and promptly starting treatment with high-dose steroids in patients presenting with AION can result in rapid blindness and poor vision recovery [1].

**Table 1:** Clinical and laboratory features of GCA GCA- giant cell arteritis; ESR- Erythrocyte sedimentation rate; CRP- C-reactive protein Ref no- [4]

Clinical Feature	Frequency (%)
Elevated ESR and/or elevated CRP	90-95
Headache	70-90
Audiovestibular manifestations (hearing loss, tinnitus, vertigo, etc.)	Up to 90
Polymyalgia rheumatica	40-60
Constitutional symptoms (fever, fatigue, or weight loss)	30-60
Abnormal temporal artery on physical examination (tenderness or absent/diminished pulses)	30-60
Jaw claudication	40-50
Scalp tenderness	33-50
Visual disturbances	20-50
Respiratory symptoms (cough, sore throat, or hoarseness)	~ 10
Cerebrovascular accidents (transient ischemic attack or stroke)	3-7
Scalp necrosis	< 5
Tongue necrosis	< 5

Although the prognosis for vision recovery in patients presenting with NAION is poor, it is better compared to the prognosis for vision recovery in patients presenting with AION. The Ischemic Optic Neuropathy Decompression Trial (IONDT) showed that 43% of patients with NAION regained at least three lines of visual acuity on the Snellen eye chart within six months. In contrast, a study by Liu et al. showed that only 34% of patients presenting with visual loss due to GCA experienced visual improvement [5,6]. While there is a 5% risk of NAION recurring in the same eye, there is a 15-20% risk of NAION occurring in the fellow eye [2,3]. NAION is often associated with risk factors like OSA, atherosclerosis, diabetes mellitus, hypertension, hyperlipidemia, smoking, and PDE-5 inhibitors, most of which were present in our patient [3]. Although there is no treatment for NAION, controlling such risk factors is paramount in the management of NAION and in decreasing the chance of NAION occurring in the other eye [2].

Obstructive sleep apnea, in particular, is strongly associated with NAION. Palombi et al. have demonstrated that OSA is the most frequent disorder associated with NAION. They found that 89% of patients newly diagnosed with NAION and underwent polysomnography had an AHI of more than 15 events per hour and that the relative risk of OSA in patients with NAION compared to the general population was 4.9 [7]. Multiple theories by which OSA is thought to contribute to the development of NAION have been proposed. One theory suggests that hypoxia caused by OSA leads to damage to the optic nerve. Another theory is that intermittent sympathetic surges caused by repeated apneic events result in impaired autoregulation of the optic nerve. Additionally, it is thought that elevated intracranial pressure during apneic events may damage the optic nerve directly or through circulatory compression [8]. OSA is often treated with CPAP therapy during sleep, and one study has shown that treating patients who have OSA with CPAP therapy decreased their probability of developing NAION [9,10].

## Conclusions

As discussed above, clinicians need to be able to distinguish NAION from AION. Additionally, clinicians should be aware of the risk factors associated with NAION, particularly OSA. By addressing such risk factors, clinicians can help prevent the development of NAION in their patients.
